# Body Fat Equations and Electrical Bioimpedance Values in Prediction of Cardiovascular Risk Factors in Eutrophic and Overweight Adolescents

**DOI:** 10.1155/2013/501638

**Published:** 2013-05-16

**Authors:** Franciane Rocha Faria, Eliane Rodrigues Faria, Roberta Stofeles Cecon, Djalma Adão Barbosa Júnior, Sylvia do Carmo Castro Franceschini, Maria do Carmo Gouveia Peluzio, Andréia Queiroz Ribeiro, Pedro Israel Cabral Lira, Paulo Roberto Cecon, Silvia Eloiza Priore

**Affiliations:** ^1^Department of Nutrition and Health-CCB II, Federal University of Viçosa, Avenida P.H. Rolfs s/n°, Campus UFV, Viçosa, MG, Brazil; ^2^Department of Rural Economy, Federal University of Viçosa, Avenida P.H. Rolfs s/n°, Campus UFV, Viçosa, MG, Brazil; ^3^Department of Nutrition-Center for Health Sciences, Federal University of Pernambuco, Avenida Nelson Chaves s/n°, Cidade Universitária, Recife, PE, Brazil; ^4^Department of Statistics, Federal University of Viçosa, Avenida P.H. Rolfs s/n°, Campus UFV, Viçosa, MG, Brazil

## Abstract

The aim of this study was to analyze body fat anthropometric equations and electrical bioimpedance analysis (BIA) in the prediction of cardiovascular risk factors in eutrophic and overweight adolescents. 210 adolescents were divided into eutrophic group (G1) and overweight group (G2). The percentage of body fat (% BF) was estimated using 10 body fat anthropometric equations and 2 BIA. We measured lipid profiles, uric acid, insulin, fasting glucose, homeostasis model assessment-insulin resistance (HOMA-IR), and blood pressure. We found that 76.7% of the adolescents exhibited inadequacy of at least one biochemical parameter or clinical cardiovascular risk. Higher values of triglycerides (TG) (*P* = 0.001), insulin, and HOMA-IR (*P* < 0.001) were observed in the G2 adolescents. In multivariate linear regression analysis, the % BF from equation (5) was associated with TG, diastolic blood pressure, and insulin in G1. Among the G2 adolescents, the % BF estimated by (5) and (9) was associated with LDL, TG, insulin, and the HOMA-IR. Body fat anthropometric equations were associated with cardiovascular risk factors and should be used to assess the nutritional status of adolescents. In this study, equation (5) was associated with a higher number of cardiovascular risk factors independent of the nutritional status of adolescents.

## 1. Background

Obesity is a proinflammatory state involving the hypertrophy and hyperplasia of adipocytes related to metabolic and cardiovascular diseases. In recent years, it has become evident that adipose tissue is not a passive receptacle of lipids, but a dynamic organ involved in the process of obesity, type 2 diabetes mellitus, hypertension, atherosclerosis, dyslipidemia, acute and chronic inflammatory processes, and metabolic syndrome, among others [1].

Obesity is a chronic, complex, multifactorial disease resulting from the interaction of genetics, environment, and lifestyle, and it is considered a public health problem worldwide due to its increasing prevalence in recent decades in developed and developing countries [2].

Obesity in adolescence tends, in 80% of cases, to perpetuate in adulthood and it is associated with high mortality [3]. Excess body fat, mainly located in the abdominal region, is a major contributor to the metabolic changes of carbohydrates and lipids, metabolic syndrome, hypertension, and coronary artery disease due to its high lipolytic capacity, decreased sensitivity antipolytic activity of insulin, and secretion of proinflammatory adipokines [4].

On the basis of their body mass index (BMI), normal individuals may carry a high percentage of body fat because the BMI cannot discriminate excess body fat from the fat-free mass (FFM) [5]. However, there are several equations for estimating body fat using anthropometric measurements, carried out according to sex, race, age, which can be divided into specific and generalized, validated in homogeneous and heterogeneous groups, respectively [6]. Only a few studies have combined these equations with cardiovascular risk factors in adolescence.

Identification of body fat evaluation methods that associate to higher number of cardiovascular risk factors is necessary on obesity prevention and treatment, specially during adolescence, to avoid that harmful health effect persists in future life. 

The objective of this study was to analyze the body fat values obtained by anthropometric equations and electrical bioimpedance analysis (BIA) in the prediction of cardiovascular risk factors in eutrophic and overweight adolescents of both sexes in Viçosa, Minas Gerais, Brazil.

## 2. Methods

### 2.1. Design and Subjects

 This was a cross-sectional study conducted between March and October 2010 involving adolescents of both sexes aged 15–18 years from the urban area of Viçosa, Minas Gerais, Brazil.

The sample size was calculated using software Epi Info version 6.04 based on a specific formula for cross-sectional studies. We considered the population of 5010 adolescents at the age studied, in Viçosa-Minas Gerais [18], prevalence of 50% [19], because aimed to consider as the outcome multiple cardiovascular risk factors, variability acceptable was 7% [20] and confidence level was 95%, indicating a minimum enrollment of 189 adolescents.

The participants were selected by simple random sampling according to the following inclusion criteria: to be postpubescent, for so, it was considered as criterion presence of menarche of at least 1 year for girls and axillary hair for boys; not having participated in studies/nutrition consultations in the last 6 months; to be normal weight presenting BMI/age ≥25 percentile and BMI/age ≤75 percentile or to be overweight, BMI/age ≥85 percentile [21]. Those cut points were established with the aim of characterizing two distinct study groups and avoid possible confounding factors related to anthropometrics. For so, we opted not to include adolescent with BMI/age >75 percentile and BMI/age <85 percentile, as these would be close to overweight and possibly with high % BF. 

The exclusion criteria included reports of infections or diagnosis of acute and chronic noncommunicable diseases; use of medications or supplements that might interfere with the metabolism of carbohydrates, lipids, and blood pressure; regular use of diuretics/laxatives or contraceptives for less than 2 months; use of a pacemaker or prosthesis; and pregnancy.

According to the classification of nutritional status [21], adolescents were grouped into the following.

- Group 1 (G1) (*n* = 140): normal weight, BMI/age ≥25 percentile, and BMI/age ≤75 percentile.
- Group 2 (G2) (*n* = 70): overweight, BMI/age ≥85 percentile.

Racial identification was performed using the hetero-attribution method according to phenotypic characteristics, such as hair type as observed by their appearance on the part closest to the scalp, type of nose, mouth, chin, and skin color [22], classified according to the Brazilian Institute of Geography and Statistics [23]. For analyses, black and brown-skinned participants were grouped together and termed “non-whites,” as there were no differences between black and brown skin regarding body fat measurements and biochemical and clinical parameters. The level of physical activity was classified in sedentary, irregularly active, active, or very active according to the International Physical Activity Questionnaire short version, which posses seven questions concerning vigorous and moderate physical activity, walking and sitting time [24].

The study was approved by the Ethics Committee on Human Research of the Federal University of Viçosa (Of. Ref. No. 084/2009), and all volunteers and their guardians signed an informed consent form before the study commenced.

### 2.2. Anthropometry and Body Composition

For anthropometric evaluation, weight and height were measured using the techniques proposed by the World Health Organization (WHO) [25]. Weight was measured using an electronic digital scale (Kratos-Cas, Brazil) with a maximum capacity of 150 kg and a sensitivity of 50 g, and height was measured using a portable stadiometer with a maximum length of 2.13 m and resolution of 0.1 cm (Altura Exata, Brazil). The measurements were made in duplicate, allowing researchers to derive the mean of the 2 measurements. If the difference between the measurements exceeded 0.5 cm, another measurement was obtained. Those overweight or obese were grouped together and termed “overweight.”

The brachial perimeter was measured on the right side, avoiding compression of the soft tissues, with an inextensible, 1.5 m long measuring tape divided into centimeters and subdivided into millimeters [26]. Measurements were performed in duplicate and the mean value of 2 measurements was used.

The biceps, triceps, suprailiac, subscapular, midaxillary, and calf skinfold thicknesses were measured on the right or left, with the subject in a standing or seated position, according to protocol [7–17, 26, 27]. The skinfold thicknesses were measured 3 times by a trained examiner and in the order mentioned with a Lange adipometer (Cambridge Scientific, Cambridge, MA, USA). We obtained the mean of the 2 closest values except for the measurements on the left side, for which the mean of 3 measurements was used instead, according to the protocol of Slaughter et al. [10]. 

The percentage of body fat (% BF) was estimated by tetra-polar horizontal BIA (Biodynamics 450; Biodynamics Corporation, Seattle, WA, USA) (BIA1), and tetrapolar vertical BIA using 8-point tactile electrode (InBody 230, BioSpace Inc., Los Angeles, CA, USA) (BIA2), being all participants fasted for at least 12 hours and undergoing the proposed evaluation protocol [28]; and by body fat anthropometric equations developed by Durnin and Rahaman [7] and Durnin and Womersley [8] (equation (1)); Boileau et al. (equation (2)) [9]; Slaughter et al. (equations (3) and (4)) [10]; Weststrate and Deurenberg (equation (5)) [11]; Guo et al. (equation (6)) [12]; Deurenberg et al. (equation (7)) [13]; Deurenberg et al. (equation (8)) [14]; Houtkooper et al. (equation (9)) [15]; and Ellis [16] and Ellis et al. [17] (equation (10)) (Table 1).

The resistance value provided by BIA1 was used in the predictive equation of FFM. Body fat (kg) was determined by the difference between the body weight (kg) and FFM (kg), and consequently, the value of the % BF was derived from the ratio between body fat (kg) and body weight, multiplied by 100. The % BF was classified according to Lohman [29]. Adolescents classified as overweight and at risk of overweight were grouped and categorized as those with excess body fat.

### 2.3. Biochemistry and Clinical Analysis

We measured total cholesterol (TC), high-density lipoprotein (HDL), and triglycerides (TG) by enzymatic calorimetric method and low-density lipoprotein (LDL) calculated by Friedewald's formula, once no TG value was higher than 400 mg/dL. We measured fasting glucose by glucose oxidase enzymatic method and insulin by electrochemiluminescence method.

Lipid profiles and hyperinsulinemia were classified according to the I Guideline for Prevention of Atherosclerosis in Childhood and Adolescence [30]. Fasting glucose was classified according to the American Diabetes Association [31].

Insulin resistance was calculated by homeostasis model assessment-insulin resistance (HOMA-IR). Presence of insulin resistance was defined as HOMA-IR ≥ 3.16 [30].

Uric acid was measured by enzymatic calorimetric method classified according to sex, considering adequate values of 2.0–7.0 mg/dL for boys and 1.5–6.0 mg/dL for girls.

Blood pressure was measured and analyzed according to the recommendations of the VI Brazilian Guidelines on Hypertension [32] using an automatic insufflation blood pressure monitor (Omron Model HEM-741 CINT; Omron Healthcare Inc., Lake Forest, IL, USA).

### 2.4. Statistical Analysis

 We used the Kolmogorov-Smirnov normality test to assess the type of distribution of variables, and Student's* t*-test or the Mann-Whitney test, Chi-square Pearson test, and linear regression to assess the relationship between the cardiovascular risk factors (dependent variable) and the % BF estimated by body fat anthropometric equations and BIA (independent variable), according to the study groups. The variables TG, insulin, HOMA-IR, LDL, TC and the ratios LDL/HDL and TC/HDL were submitted to logarithmic transformation, for not presenting normal distribution. The values of body fat anthropometric equations and BIA that presented coefficient *β* exhibiting significance of *P* < 0.20 in the bivariate analysis were used in multiple regression models adjusted for sex, and in G1, also for normal individuals with excess body fat. The level of physical activity, considered a confounding factor, was not included in the model because it was not associated with risk factors and % BF in the bivariate analysis.

To analyze the fit of the models, we first evaluated the normality of the distribution of residuals, which should be normally distributed, in order to examine the prerequisites of the classical linear regression model. For this, we used the Shapiro-Wilk statistic level of significance *P* > 0.05. The presence of heteroskedasticity was verified by the White test; when necessary, the consistent variance matrix modified for small samples was used to correct the estimated error pattern [33]. For multiple regression analysis, the variance inflation factor was used as an indicator of multicollinearity.

The data were double-entered in Microsoft Office Excel 2007, and statistical analyses were performed in SPSS for Windows version 13.0 and STATA version 11.0, with a significance level of *P* < 0.05.

## 3. Results

The study evaluated 210 adolescents, mean age was 16.8 (±1.0) years, 52.4% (*n* = 110) were women, 61.4% (*n* = 129) were non-whites, 51% (*n* = 107) were actives, 76.7% (*n* = 161) exhibited inadequacy of at least one biochemical parameter or clinical cardiovascular risk, 44.8% (*n* = 94) had TC ≥150 mg/dL, 24.3% (*n* = 51) had LDL ≥100 mg/dL, 43.3% (*n* = 91) had HDL ≤45 mg/dL, 15.2% (*n* = 32) had TG ≥100 mg/dL, 1% (*n* = 2) had uric acid >7 mg/dL, and 3.9% (*n* = 8) had systolic or diastolic blood pressure ≥120/80 mmHg. Higher values of TG (*P* = 0.001), TC/HDL (*P* = 0.001), LDL/HDL (*P* = 0.005), insulin, and HOMA-IR (*P* < 0.001) were observed in the G2 adolescents (Table 2), and regarding physical activity level boys were more active (*P* < 0.05).

After analyzing only the G1 adolescents, we found that they had excess body fat of 10.7–82.1% according to the body fat anthropometric equations or BIA, and 92.1% (*n* = 129) of these adolescents had excess adiposity according to least one method of body composition assessment, being more frequent in feminine sex (*P* < 0.001).

In the simple linear regression analysis for the G1 adolescents, the % BF as estimated by anthropometric equations and BIA was associated with cardiovascular risk factors, except HDL, TC/HDL, and LDL/HDL which were not associated with the % BF as estimated from these methods. Likewise, no association was found for the % BF obtained from (6), (8), and (10) for TG and from (4) for LDL (Table 3). However, when fitting the model according to sex and eutrophic individuals with excess body fat (false negatives), we observed that the associations between the % BF from the 10 equations and 2 BIA and TC, LDL, systolic blood pressure (SBP), and fasting glucose did not remain significant. In multiple regression analysis, only the % BF from (5) remained associated with more than one risk factor, in this case, TG, diastolic blood pressure (DBP), and insulin, the latter with a correlation coefficient of 0.51, which shows that 51% of the changed insulin levels in eutrophic adolescents were explained by the % BF as estimated by the proposal of Weststrate and Deurenberg (equation (5)) (Table 4).

In the G2 adolescents, the majority of the % BF was associated with the dependent variables TC, HDL, insulin, HOMA-IR, and DBP (Table 5). No association was found between the estimates of % BF and SBP, TC/HDL, and only the % BF obtained from equation (3) was associated with fasting glucose (*P* < 0.05); however, when adjusting the model by sex, this association did not remain significant (Tables 5 and 6). With the exception of equation (10) for DBP, the associations between the % BF and biochemical parameters HDL, glucose, and DBP did not remain significant in the multiple regression analysis. All % BF values obtained in the study maintained their association with TG, insulin, and HOMA-IR, which are components of the metabolic syndrome, with *R* ^2^ ranging from 0.07 to 0.19 depending on the parameter evaluated. There were higher coefficients of determination in the associations between body fat and uric acid; 39% of the change of this parameter in the overweight adolescents, regardless of sex, was explained by the % BF estimated by equation (10) (Table 6).

Considering the number of risk factors associated with the % BF, we found that the best equation for the G1 adolescents was equation (5), and (9) was the best suited for the G2 adolescents.

## 4. Discussion

The results of this study are broadly in agreement with the literature [34–36] that reveals the presence of cardiovascular risk factors in adolescents, especially as related to inappropriate lipid profile and high body fat percentage. The BMI alone cannot determine the nutritional status of overweight or obese adolescents, limiting its exclusive use [5], for according to the present study, normal individuals by BMI/age, may carry excess body fat and are metabolically similar to those carrying excess weight [34].

As expected, the frequency of excess adiposity varied according to the body fat anthropometric equations and BIA, possibly due to the peculiarities of each method, which involves characteristics related to the population, age, race, and nutritional status in addition to anthropometric measurements used as independent variables. Excess body fat was more frequent in girls, which may in part be explained by the differences in body composition between the sexes. Although weight gain is also a result of increased muscle mass and adipose tissue in both sexes during puberty, the gain in muscle mass is higher in boys and that for adipose tissue is higher in girls [37]. Another factor evaluated in this study that may have contributed to this higher frequency of excess adiposity in girls refers to the lowest level of physical activity when compared to boys.

As in other studies [34–36], overweight individuals had higher serum concentrations of TG, insulin, and HOMA-IR, the criteria used in the diagnosis of the metabolic syndrome as proposed by the WHO [38]. Our results are similar to those of Freedman et al. [35], who found that 58% of obese individuals possessed at least one cardiovascular risk factor, and were 7.1 times more likely to have abnormal TG levels than eutrophic individuals were.

Conversely, there was no difference in blood pressure when comparing G1 and G2 individuals, which was contrary to the findings of Falaschetti et al. [36], who assessed children and prepubertal adolescents in the United Kingdom and demonstrated that overweight individuals were 3 times more likely to have altered blood pressure levels compared to those of normal weight. The present study results may be explained partly by the fact that almost all eutrophic adolescents, according to BMI/age, carry excess body fat, that is, are false negatives, therefore are metabolically similar to overweight or obese individuals. Confirming this hypothesis, Serrano et al. [34] identified the influence of body fat on blood pressure, once there was a difference in blood pressure level between eutrophic adolescents with appropriate and high percentage of body fat, but there was no difference between this last group and overweight adolescents.

The body fat anthropometric equations and BIA evaluated in this study as a method of estimating the % BF are generally associated with cardiovascular risk factors and are good predictors of changes in TG, insulin, and HOMA-IR, mainly in overweight individuals.

These results are relevant and are consistent with the literature, which has reported that metabolic and hemodynamic changes are more frequent in obese subjects [34, 38] and that insulin resistance is the link between central obesity distribution, glucose intolerance, hypertension, dyslipidemia, coagulation disorders, hyperuricemia, and microalbuminuria, risk factors also found in metabolic syndrome [39].

According to the present study results, the % BF obtained by body fat anthropometric equations was a better predictor of cardiovascular risk than that estimated by BIA for both study groups, especially G1, as multiple regression analysis determined that did not exist associations between the % BF estimated by BIA and the cardiovascular risk parameters.

The multiple linear regression analysis performed in the G1 group revealed that the association of the % BF with TG, insulin, HOMA-IR, and DBP remained significant, indicating the presence of cardiovascular risk factors even in individuals with adequate nutritional status. These results are suggestive of the influence of other factors such as high-energy density diet, physical inactivity, and heredity, among others, that may be involved in the changes within these parameters [40].

In this study, equation (5) was associated with a greater number of cardiovascular risk factors in the G1. Similarly, in the study by Burrows et al. [41], equation (5) exhibited a higher correlation with insulin sensitivity (*r* = − 0.576, *P* < 0.0001). This result can be explained partly by the fact that, unlike the other authors whose equations are analyzed in this work, Weststrate and Deurenberg [11] developed an equation using age and body density for estimating the % BF, variables also associated to the estimative of FFM. According to the literature, FFM density varies with age, sex, ethnic group, and body fat level and physical activity, depending primarily on the proportion of water and minerals included in the FFM [26].

With regard to the G2 adolescents, equation (9) explained 5 of 7 risk factors, followed by (10) and (5). Equation (9) for the FFM differed from the others in that it had as independent variables height, weight, and resistance, which was determined by the BIA equipment and can be related with the best results. Another factor that may explain its association with risk factors is the fact that this equation be specific for adolescents [15]. It is noteworthy that the use of this equation would be restricted to research or services that provide BIA equipment to determine resistance. 

Although BIA to be a fast method, of easy utilization, noninvasive, and in this study the body fat estimated by equation (9) was associated with expressive number of cardiovascular risk factors, it is worth noticing this method tends to underestimate % BF, specially in subjects with body fat excess [42], not presenting good prediction power in these cases. 

In order to encourage the use of the body fat anthropometric equations in routine healthcare and research, we suggest the use of equations (10) and (5). The advantages of equation (10) are its simplicity and ease of collection of the variables weight, height, and age, and the measurements and information obtained are on an outpatient basis and available to most research efforts; however, this equation was better associated with cardiovascular risk factors only in overweight adolescents (i.e., G2).

Although involving more complex calculations and obtainment of measurements because it uses skinfolds thicknesses, equation (5) has the advantage of being the equation that was most associated with cardiovascular risk factors in both study groups, which means it can be used regardless of nutritional status.

In this study, we found that none of the estimates of body fat was associated with HDL, demonstrating that the variation of this parameter is independent of adiposity, being regular physical activity, dietary habits, and family history of diseases being some of the factors associated with its modification [43, 44].

Many studies have associated cardiovascular risk factors with the BMI, waist circumference, hip circumference, waist/hip and waist/height ratio, and the sum of skinfolds thicknesses as central and peripheral body fat measures, and other parameters [36], but literature regarding the comparison of body composition using body fat anthropometric equations and their association with cardiovascular risk factors is scarce, which justifies the relevance of this work.

## 5. Conclusions

 The present study results are in agreement with the literature and demonstrate that excess adiposity is associated with cardiovascular risk factors, especially those related to metabolic syndrome. As expected, excess adiposity varied according to the anthropometric equations and BIA used in this study, being more prevalent in girls.

Broadly, body fat anthropometric equations and BIA were capable of predicting cardiovascular risk factors in postpubertal adolescents, especially for overweight subjects (G2), once it was verified associations of all equations and BIA to at least one risk factor in this group.

From the results obtained and considering that all body fat anthropometric equations are differentiated by sex, it can be inferred that, in this study, the best anthropometric equation was the one of Weststrate and Deurenberg (equation 5), as it was associated with a higher number of cardiovascular risk factors independently of the nutritional status of adolescents.

Considering the increasing prevalence of overweight/obesity and cardiovascular risk factors in adolescents, the identification of a noninvasive and inexpensive method, as is the case with body fat anthropometric equations, permits the early detection of excess body fat, allowing for interventions in a period conducive to the reduction of such risk factors.

## Figures and Tables

**Table 1:** Body fat and fat-free mass anthropometric equations.

Reference	Sex	Equation	Equation number
	Masc.^a^	*D* = 1.1533 – 0.0643 [log*∑*4 skinfold thicknesses]	
Durnin and Rahaman (1967) [7]	Fem.^a^	*D* = 1.1369 – 0.0598 [log*∑*4 skinfold thicknesses]	
		% BF = [(4.95/*D*) – 4.50] × 100	(1)
	Masc.^b^	*D* = 1.1620 – 0.063 [log*∑*4 skinfold thicknesses]	
Durnin and Womersley (1974) [8]	Fem.^b^	*D* = 1.1549 – 0.0678 [log*∑*4 skinfold thicknesses]	
		% BF = [(4.95/*D*) – 4.50] × 100	

	Masc.	% BF = 1.35 [triceps (mm) + subscapular (mm)] – 0.012	
Boileau et al. (1985) [9]		[triceps (mm) + subscapular (mm)]^2^ – 4.4	(2)
	Fem.	% BF = 1.35 [triceps (mm) + subscapular (mm)] – 0.012	
		[triceps (mm) + subscapular (mm)]^2^ – 2.4	

	Masc. Fem.	% BF = 0.735 [triceps (mm) + calf (mm)] + 1.0 % BF = 0.610 [triceps (mm) + calf (mm)] + 5.1	(3)
	Masc.^c^	% BF = 1.21 [triceps (mm) + subscapular (mm)] – 0.008	
Slaughter et al. (1988) [10]		[triceps (mm) + subscapular (mm)]^2^ + *I*	
	Fem.^c^	% BF = 1.33 [triceps (mm) + subscapular (mm)] – 0.013	(4)
		[triceps (mm) + subscapular (mm)]^2^ – 2.5	
	Masc.^d^ Fem.^d^	% BF = 0.783 [triceps (mm) + subscapular (mm)] + 1.6 % BF = 0.546 [triceps (mm) + subscapular (mm)] + 9.7	

Weststrate and Deurenberg (1989) [11]	Masc. Fem.	% BF = ({562 − 4.2[*A* − 2]}/*D* ^†^ − {525 − 4.7[*A* − 2]} % BF = ({553 − 7.3[*A* − 10]}/*D* ^†^ − {514 − 8.0[*A* − 10]}	(5)

	Masc.	FFM = 0.646 (Wt) – 0.116 [lateral calf (mm)] – 0.375	
Guo et al. (1989) [12]		[midaxillary (mm)] + 0.475 [arm muscle circumference (cm)] + 0.156 (Ht^2^/*R*) – 2.932	(6)
	Fem.	FFM = 0.682 (Wt) – 0.185 [lateral calf (mm)] – 0.244	
		[triceps (mm)] – 0.202 [subscapular (mm)] + 0.182 (Ht^2^/*R*) + 4.338	

Deurenberg et al. (1990) [13]	Masc. Fem.	% BF = 18.88 [log (*∑*4 skinfold thicknesses)] – 15.58 % BF = 39.02 [log (*∑*4 skinfold thicknesses)] – 43.49	(7)

Deurenberg et al. (1991) [14]	Masc./Fem.^e^ Masc. /Fem.^f^	% BF = 1.51 (BMI) – 0.70 (*A*) – 3.6 (*S*) + 1.4 % BF = 1.2 (BMI) + 0.23 (*A*) – 10.8 (*S*) −5.4	(8)

Houtkooper et al. (1992) [15]	Masc./Fem.	FFM = 0.61 (Ht^2^/*R*) + 0.25 (Wt) + 1.31	(9)

Ellis (1997) [16] and Ellis et al. (1997) [17]	Masc.^g^ Masc.^h^ Fem.^g^ Fem.^h^	*F* = 0.534 (Wt) – 1.59 (*A*) + 3.03 *F* = 0.594 (Wt) – 0.381 (Ht) + 36.0 *F* = 0.642 (Wt) – 0.120 (Ht) – 0.606 (*A*) + 8.98 *F* = 0.653 (Wt) – 0.163 (Ht) – 0.298 (*A*) + 10.7	(10)

^a^Equation for adolescents aged 15-16 years; ^b^equation for adolescents aged 17-18 years; ^c ^for a sum of triceps and subscapular skinfold thicknesses <35 mm; ^d^for a sum of triceps and subscapular skinfold thicknesses >35 mm; ^e^equation for adolescents aged ≤15 years; ^f^equation for adolescents aged ≥16 years; ^g^equations for white individuals; ^h^equations for black individuals; log*∑*4 skinfold thicknesses = logarithm sum of bicipital, tricipital, suprailiacal, subscapular skinfold thicknesses (mm); for boys aged 2–18 y: *D* ^†^ = {1.1315 + (0.0018[*A* − 2])} − {0.0719 − 0.0006[*A* − 2] × log(∑4  skin  fold  thicknesses)} and for girls aged 11–18 y: *D* ^†^ ={1.1350 + (0,0031[*A* − 10])} − {0.0719 − 0.0003[*A* − 2] × log(∑4  skin  fold  thicknesses)}; Eq: equation; Fem: feminine sex; Masc.: masculine sex; *D*: density (g/L); % BF: percentage of body fat; FFM: fat free mass (kg), *R*: resistance (Ω); *A*: age (years); *F*: fat (kg); BMI: body mass index (kg/m^2^); *S*: sex (masculine = 1 and feminine = 0); Wt: body weight (kg); Ht: height (cm); *I*: intercept in males varies for maturation level and racial group as follows: for black males: prepubescent = − 3.5; pubescent = − 5.2; postpubescent and adult = − 6.8. For white males: prepubescent = − 1.7; pubescent = − 3.4; postpubescent and adult = − 5.5.

**Table 2:** Physical and metabolic characteristics of the adolescents according to studied group (Viçosa, Minas Gerais, Brazil (2010)).

Parameters	Group				
	G1		G2		*P* value
	Mean (SD)	Median (Min–Max)	Mean (SD)	Median (Min–Max)
Age (years)	16.9 ± 1.0	16.9 (15.1–19.0)	16.7 ± 1.0	18.9 (15.0–18.7)	0.910^a^
Weight (kg)	60.2 ± 6.1	59.8 (44.7–75.9)	75.7 ± 13.7	72.9 (57.5–128.7)	<0.0001^b^
Height (cm)	168.4 ± 8.2	168.5 (149.5–185.7)	166.4 ± 10.3	165.4 (149.2–191.3)	0.021^a^
BMI (kg/m^2^)	21.2 ± 1.1	21.3 (18.7–23.6)	27.2 ± 3.0	26.4 (23.1–40.1)	<0.0001^b^
Fasting glucose (mg/dL)	84.0 ± 6.0	84.0 (69.0–99.0)	84.7 ± 7.0	85.0 (71.0–98.0)	0.082^a^
TC (mg/dL)	146.6 ± 26.3	146.0 (83.0–271.0)	152.0 ± 28.3	149.0 (87.0–252.0)	0.945^a^
HDL (mg/dL)	48.9 ± 11.0	48.0 (21.0–74.0)	44.8 ± 10.7	43.0 (27.0–72.0)	0.799^a^
LDL (mg/dL)	85.1 ± 22.9	85.3 (44.8–202.8)	90.0 ± 22.4	88.2 (40.2–148.6)	0.819^a^
TC/HDL	3.11 ± 0.75	3.04 (1.88–6.57)	3.54 ± 0.93	3.44 (1.96–6.63)	0.001^b^
LDL/HDL	1.83 ± 0.63	1.75 (0.76–4.39)	2.12 ± 0.73	2.05 (0.77–4.02)	0.005^b^
TG (mg/dL)	63.2 ± 26.9	60.0 (24.0–189.0)	85.2 ± 53.3	76.0 (31.0–320.0)	0.0001^b^
Uric Acid (mg/dL)	3.7 ± 1.1	3.7 (1.5–7.4)	3.8 ± 1.2	3.7 (1.6–7.5)	0.980^a^
Insulin (*μ*U/dL)	9.1 ± 3.6	9.0 (2.0–23.4)	12.8 ± 5.6	11.2 (3.9–27.1)	<0.0001^b^
HOMA-IR	1.9 ± 0.8	1.8 (0.4–4.6)	2.7 ± 1.3	2.3 (0.8–6.4)	0.0001^b^
SBP (mmHg)	102.9 ± 8.2	102.8 (82.0–125.0)	104.7 ± 9.3	104.0 (83.0–125.0)	0.083^a^
DBP (mmHg)	60.5 ± 7.9	61.5 (40.0–84.0)	61.1 ± 8.7	61.0 (43.0–82.0)	0.592^a^

^a^Student's *t*-test; ^b^Mann-Whitney test, SD: standard deviation, Min: minimum, Max: maximum, BMI: body mass index, TC: total cholesterol, HDL: high-density lipoprotein, LDL: low-lipoprotein density, TG: triglycerides, HOMA-IR: homeostasis model assessment-insulin resistance, SBP: systolic blood pressure, DBP: diastolic blood pressure.

**Table 3:** Simple linear regression coefficient (*β*) and coefficient of determination (*r* ^2^) of the relationship between percentage of body fat and biochemical and clinical parameters in eutrophic adolescents (G1) (Viçosa, Minas Gerais, Brazil (2010)).

		Eq (1)	Eq (2)	Eq (3)	Eq (4)	Eq (5)	Eq (6)	Eq (7)	Eq (8)	Eq (9)	Eq (10)	BIA1	BIA2
Fasting glucose (mg/dL)	*β*	−0.23^‡^	−0.27^‡^	−0.26**	−0.21**	−0.26^‡^	−0.19^‡^	−0.26^‡^	−0.44^‡^	−0.25^‡^	−0.26^‡^	0.25^‡^	−0.22^‡^
	*r* ^2^	0.09	0.10	0.08	0.08	0.10	0.12	0.13	0.14	0.09	0.12	0.10	0.09
TC (mg/dL)	*β*	0.003^‡^	0.004^‡^	0.004^‡^	0.003**	0.003^‡^	0.002**	0.003**	0.004**	0.002**	0.003**	0.002**	0.002**
	*r* ^2^	0.09	0.11	0.09	0.08	0.08	0.07	0.08	0.07	0.05	0.08	0.06	0.05
HDL (mg/dL)	*β*	0.09	0.15	0.13	0.09	0.10	0.08	0.13	0.24	0.08	0.15	0.10	0.06
	*r* ^2^	0.004	0.009	0.005	0.005	0.004	0.006	0.01	0.01	0.003	0.01	0.005	0.002
LDL (mg/dL)	*β*	0.004**	0.005**	0.004**	0.004	0.004**	0.003**	0.003**	0.005**	0.003*	0.004**	0.003*	0.003*
	*r* ^2^	0.07	0.08	0.06	0.07	0.07	0.06	0.06	0.05	0.04	0.07	0.05	0.05
TG (mg/dL)	*β*	0.006**	0.006**	0.008^‡^	0.004*	0.006**	0.003	0.004*	0.004	0.004*	0.003	0.005*	0.004*
	*r* ^2^	0.06	0.06	0.09	0.04	0.06	0.04	0.04	0.02	0.03	0.02	0.04	0.04
TC/HDL	*β*	0.002	0.002	0.002	0.001	0.002	0.001	0.001	0.002	0.002	0.001	0.002	0.002
	*r* ^2^	0.02	0.02	0.02	0.02	0.02	0.01	0.01	0.006	0.02	0.01	0.02	0.02
LDL/HDL	*β*	0.003	0.003	0.003	0.002	0.003	0.002	0.002	0.003	0.003	0.002	0.002	0.002
	*r* ^2^	0.03	0.02	0.02	0.02	0.02	0.02	0.01	0.001	0.02	0.02	0.01	0.02
Uric Acid (mg/dL)	*β*	−0.08^‡^	−0.09^‡^	−0.09^‡^	−0.08^‡^	−0.09^‡^	−0.07^‡^	−0.08^‡^	−0.14^‡^	−0.09^‡^	−0.09^‡^	−0.09^‡^	−0.08^‡^
	*r* ^2^	0.31	0.31	0.26	0.33	0.32	0.39	0.38	0.39	0.29	0.40	0.35	0.32
Insulin (*μ*U/dL)	*β*	−0.1^‡^	−0.01^‡^	−0.1^‡^	−0.1^‡^	−0.01^‡^	−0.008^‡^	−0.01^‡^	−0.02^‡^	−0.01^‡^	−0.01^‡^	−0.01^‡^	−0.01^‡^
	*r* ^2^	0.31	0.31	0.25	0.33	0.31	0.39	0.37	0.39	0.28	0.40	0.34	0.31
HOMA-IR	*β*	0.008^‡^	0.009^‡^	0.01^‡^	0.006**	0.008^‡^	0.005**	0.006**	0.008*	0.008^‡^	0.004*	0.008^‡^	0.006**
	*r* ^2^	0.10	0.09	0.13	0.07	0.10	0.08	0.08	0.04	0.08	0.04	0.09	0.08
SBP (mmHg)	*β*	−0.36^‡^	−0.35^‡^	−0.36**	−0.32^‡^	−0.35^‡^	−0.28^‡^	−0.34^‡^	−0.56^‡^	−0.32**	−0.31	−0.37^‡^	−0.33^‡^
	*r* ^2^	0.12	0.09	0.08	0.09	0.09	0.13	0.12	0.13	0.08	0.09	0.12	0.11
DBP (mmHg)	*β*	0.25**	0.34**	0.33**	0.30^‡^	0.33^‡^	0.20**	0.27**	0.36**	0.32**	0.28**	0.26**	0.27**
	*r* ^2^	0.06	0.09	0.07	0.09	0.09	0.07	0.08	0.05	0.08	0.08	0.06	0.08

BIA1: tetra-polar horizontal electrical bioimpedance analysis; BIA2: tetra-polar vertical electrical bioimpedance analysis using 8-point tactile electrode; Eq: equation; TC: total cholesterol; TG: triglycerides; HDL: high-density lipoprotein; LDL: low-density lipoprotein; HOMA-IR: homeostasis model assessment-insulin resistance; SBP: systolic blood pressure; DBP: diastolic blood pressure; *β*: coefficient of linear regression; *r* ^2^: coefficient of determination; *P < 0.05; **P < 0.001; ^‡^ *P* < 0.0001.

**Table 4:** Multiple linear regression coefficient (*β*) and coefficient of determination (*R* ^2^) of the relationship between percentage of body fat, adjusted for sex and normal individuals with excess body fat, and biochemical and clinical parameters in eutrophic adolescents (G1) (Viçosa, Minas Gerais, Brazil (2010)).

		Eq (1)	Eq (2)	Eq (3)	Eq (4)	Eq (5)	Eq (6)	Eq (7)	Eq (8)	Eq (9)	Eq (10)	BIA1	BIA2
Fasting glucose (mg/dL)	*β*	0.21	0.11	—	0.21	0.28	0.11	0.13	−0.29	0.08	—	0.22	0.25
	*R* ^2^	0.18	0.17		0.18	0.18	0.17	0.17	0.19	0.17		0.17	0.18
TC (mg/dL)	*β*	0.002	0.003	0.003	0.0007	0.004	−0.002	0.001	−0.001	−0.003	0.002	0.0001	−0.003
	*R* ^2^	0.09	0.11	0.10	0.09	0.10	0.11	0.08	0.08	0.09	0.09	0.08	0.10
LDL (mg/dL)	*β*	0.004	0.004	0.003	0.003	0.007	−0.0007	0.003	−0.0005	−0.001	0.004	0.0008	−0.002
	*R* ^2^	0.07	0.08	0.07	0.07	0.08	0.07	0.06	0.06	0.06	0.07	0.06	0.07
TG (mg/dL)	*β*	—	0.02^‡^	0.02^‡^	0.008	0.02^‡^	—	—	—	−0.004	—	0.01	0.001
	*R* ^2^		0.11	0.12	0.04	0.11				0.08		0.05	0.06
Insulin (*μ*U/dL)	*β*	—	—	0.003	0.003	0.01**	—	—	—	—	—	0.001	0.003
	*R* ^2^			0.49	0.49	0.51						0.49	0.49
HOMA-IR	*β*	—	—	—	0.01**	—	—	—	—	—	—	—	—
	*R* ^2^				0.09								
SBP (mmHg)	*β*	0.03	0.24	0.002	0.13	0.33	−0.02	0.14	0.01	0.22	0.43	−0.17	−0.02
	*R* ^2^	0.15	0.15	0.15	0.15	0.16	0.15	0.15	0.15	0.15	0.18	0.15	0.15
DBP (mmHg)	*β*	−0.01	0.16	0.20	0.37	0.74**	0.11	0.36	−0.13	0.29	0.14	0.19	0.28
	*R* ^2^	0.05	0.09	0.07	0.09	0.12	0.08	0.08	0.05	0.08	0.09	0.07	0.06

*Note*. HDL, TC/HDL, LDL/HDL, and uric acid did not meet the assumptions of linear regression. BIA1: tetra-polar horizontal electrical bioimpedance analysis; BIA2: tetra-polar vertical electrical bioimpedance analysis using 8-point tactile electrode; Eq: equation; TC: total cholesterol; TG: triglycerides; LDL: low-density lipoprotein; HOMA-IR: homeostasis model assessment-insulin resistance; SBP: systolic blood pressure; DBP: diastolic blood pressure; *β*: coefficient of multiple linear regression, *R* ^2^: coefficient of determination, —: did not meet the assumptions of linear regression; *P < 0.05; **P < 0.001; ^‡^ *P* < 0.0001.

**Table 5:** Simple linear regression coefficient (*β*) and coefficient of determination (*r* ^2^) of the relationship between percentage of body fat and biochemical and clinical parameters in overweight adolescents (G2) (Viçosa, Minas Gerais, Brazil (2010)).

		Eq (1)	Eq (2)	Eq (3)	Eq (4)	Eq (5)	Eq (6)	Eq (7)	Eq (8)	Eq (9)	Eq (10)	BIA1	BIA2
Fasting glucose (mg/dL)	*β*	0.19	0.13	0.16*	0.17	0.22	0.08	0.04	0.15	0.13	0.13	0.18	0.06
	*r* ^2^	0.03	0.01	0.06	0.005	0.03	0.01	0.002	0.02	0.01	0.02	0.03	0.005
TC (mg/dL)	*β*	0.004**	0.0003	0.002**	0.004^‡^	0.006**	0.003**	0.003**	0.004**	0.006^‡^	0.004**	0.005	0.004
	*r* ^2^	0.12	0.00	0.11	0.17	0.16	0.14	0.12	0.11	0.18	0.12	0.16	0.17
HDL (mg/dL)	*β*	0.41*	0.58*	0.08	0.25	0.47*	0.37**	0.42**	0.34	0.56*	0.46*	0.46*	0.30
	*r* ^2^	0.06	0.06	0.006	0.04	0.06	0.11	0.11	0.04	0.08	0.09	0.08	0.04
LDL (mg/dL)	*β*	0.74	−0.36	0.50*	0.62*	1.03*	0.46	0.50	0.81	1.08*	0.63	0.80	0.87*
	*r* ^2^	0.04	0.01	0.06	0.06	0.07	0.04	0.04	0.05	0.07	0.04	0.06	0.09
TG (mg/dL)	*β*	0.007	−0.002	0.006**	0.007*	0.01*	0.004	0.003	0.006	0.01*	0.004	0.008*	0.006
	*r* ^2^	0.04	0.001	0.10	0.08	0.06	0.03	0.01	0.02	0.06	0.02	0.06	0.04
TC/HDL	*β*	0.000	−0.006	0.001	0.001	0.001	−0.001	−0.001	0.001	0.0002	−0.001	0.000	0.001
	*r* ^2^	0.000	0.05	0.02	0.003	0.001	0.005	0.005	0.002	0.0001	0.002	0.000	0.006
LDL/HDL	*β*	−0.006	−0.04*	0.004	−0.002	−0.002	−0.009	−0.008	0.002	−0.006	−0.008	−0.008	0.004
	*r* ^2^	0.003	0.06	0.04	0.0004	0.0004	0.01	0.01	0.0002	0.002	0.006	0.005	0.002
Uric Acid (mg/dL)	*β*	−0.02	−0.07*	0.03*	0.01	−0.02	−0.03*	−0.05**	−0.02	−0.03	−0.04*	−0.03	−0.01
	*r* ^2^	0.01	0.06	0.08	0.01	0.009	0.07	0.13	0.02	0.02	0.06	0.02	0.005
Insulin (*μ*U/dL)	*β*	0.008*	0.01**	0.006*	0.008**	0.01*	0.004	0.004	0.01*	0.01**	0.008*	0.01**	0.006*
	*r* ^2^	0.07	0.11	0.09	0.13	0.09	0.05	0.02	0.09	0.01	0.08	0.10	0.06
HOMA-IR	*β*	0.01*	0.02**	0.006**	0.009**	0.01*	0.005	0.004	0.01*	0.01*	0.009*	0.01**	0.006
	*r* ^2^	0.08	0.10	0.10	0.13	0.09	0.05	0.02	0.08	0.09	0.07	0.10	0.05
SBP (mmHg)	*β*	−0.14	−0.28	0.04	−0.003	−0.11	−0.18	−0.14	−0.14	−0.38	−0.08	−0.29	−0.27
	*r* ^2^	0.009	0.02	0.002	0.00	0.004	0.04	0.02	0.009	0.05	0.004	0.04	0.05
DBP (mmHg)	*β*	0.38*	0.14	0.10	0.19	0.43*	0.24*	0.36**	0.54**	0.55**	0.55^‡^	0.45**	0.42**
	*r* ^2^	0.07	0.005	0.10	0.04	0.08	0.07	0.12	0.14	0.12	0.18	0.12	0.14

BIA1: tetra-polar horizontal electrical bioimpedance analysis; BIA2: tetra-polar vertical electrical bioimpedance analysis using 8-point tactile electrode; Eq: equation, TC: total cholesterol; TG: triglycerides; HDL: high-density lipoprotein; LDL: low-density lipoprotein; HOMA-IR: homeostasis model assessment-insulin resistance; SBP: systolic blood pressure; DBP: diastolic blood pressure; *β*: coefficient of linear regression; *r* ^2^: coefficient of determination; *P < 0.05; **P < 0.001; ^‡^ *P* < 0.0001.

**Table 6:** Multiple linear regression coefficient (*β*) and coefficient of determination (*R* ^2^) of the relationship between percentage of body fat, adjusted for sex, and biochemical and clinical parameters in overweight adolescents (G2) (Viçosa, Minas Gerais, Brazil (2010)).

		Eq (1)	Eq (2)	Eq (3)	Eq (4)	Eq (5)	Eq (6)	Eq (7)	Eq (8)	Eq (9)	Eq (10)	BIA1	BIA2
Fasting glucose (mg/dL)	*β*	—	—	0.16	—	—	—	—	—	—	—	—	—
	*R* ^2^			0.06									
TC (mg/dL)	*β*	0.004	−0.002	—	—	—	0.004*	—	0.004	0.007**	0.005	—	0.004**
	*R* ^2^	0.12	0.09				0.14		0.11	0.18	0.12		0.17
HDL (mg/dL)	*β*	−0.20	0.23	—	—	−0.12	0.06	−0.24	−0.34	0.09	−0.16	−0.02	—
	*R* ^2^	0.15	0.15			0.15	0.14	0.15	0.16	0.15	0.15	0.14	
LDL (mg/dL)	*β*	1.17	−1.23	2.27	0.10	1.65*	1.03	1.72	—	1.86*	0.93	2.28*	—
	*R* ^2^	0.05	0.04	0.12	0.13	0.08	0.06	0.07		0.09	0.05	0.12	
TG (mg/dL)	*β*	—	—	0.007**	0.007*	0.02**	—	—	—	0.02**	—	0.02*	—
	*R* ^2^			0.11	0.08	0.12				0.11		0.13	
Uric Acid (mg/dL)	*β*	—	−0.007	—	—	—	0.08**	0.13**	—	—	0.12**	—	—
	*R* ^2^		0.26				0.37	0.37			0.39		
Insulin (*μ*U/dL)	*β*	0.02**	—	0.006*	0.008**	0.02**	—	—	0.02**	0.02**	0.02**	0.02**	0.009*
	*R* ^2^	0.11		0.09	0.13	0.14			0.13	0.13	0.17	0.17	0.07
HOMA-IR	*β*	0.02**	—	0.006*	0.009**	0.02**	—	—	0.02**	0.02**	0.02**	—	0.009*
	*R* ^2^	0.12		0.10	0.13	0.14			0.12	0.12	0.16		0.07
DBP (mmHg)	*β*	0.05	—	—	—	0.11	−0.03	0.27	0.40	0.34	0.66*	0.28	0.31
	*R* ^2^	0.11				0.11	0.11	0.12	0.15	0.14	0.19	0.13	0.15

*Note*. TC/HDL, LDL/HDL, and SBP did not meet the assumptions of linear regression. BIA1: tetra-polar horizontal electrical bioimpedance analysis; BIA2: tetra-polar vertical electrical bioimpedance analysis using 8-point tactile electrode; Eq: equation; TC: total cholesterol; TG: triglycerides; HDL: high-density lipoprotein; LDL: low-density lipoprotein; HOMA-IR: homeostasis model assessment-insulin resistance; DBP: diastolic blood pressure; *β*: coefficient of linear regression; *R* ^2^: coefficient of determination; —: did not meet the assumptions of linear regression; *P < 0.05; **P < 0.001; ^‡^ *P* < 0,0001.
